# Mir-208 promotes cell proliferation by repressing SOX6 expression in human esophageal squamous cell carcinoma

**DOI:** 10.1186/1479-5876-12-196

**Published:** 2014-07-15

**Authors:** Heping Li, Dayong Zheng, Bing Zhang, Liangshuai Liu, Junwei Ou, Wei Chen, Shiqiu Xiong, Yong Gu, Jianyong Yang

**Affiliations:** 1 Department of Medical Imaging, the First Affiliated Hospital of Sun Yat-sen University, Guangzhou 510080, P.R. China; 2 Department of Oncology, the First Affiliated Hospital of Sun Yat-sen University, Guangzhou 510080, P.R. China; 3 Department of Oncology, Nanfang Hosptial, Southern Medical University, Guangzhou 510515, P.R. China; 4 Department of Thoracic Surgery, the First Affiliated Hospital of Sun Yat-sen University, Guangzhou 510080, P.R. China; 5 Department of Biochemistry, University of Leicester, Leicester, UK

**Keywords:** miR-208, SOX6, Esophageal squamous cell carcinoma, Proliferation

## Abstract

**Background:**

Esophageal squamous cell carcinoma (ESCC) is the major histological type of esophageal cancer in developing countries. The prognosis and survival rate of ESCC are very poor. Recently, microRNAs (miRNAs) have emerged as important regulators of cancer cell biological processes. To better understanding the molecular mechanisms by which they regulate the behavior of cancer cells is needed.

**Methods:**

The expression of miR-208 was examined in ESCC cell lines and tumor tissues by real-time PCR. Proliferation capability of ESCC cells upon regulation of miR-208 expression was detected by MTT assay, colony formation assay, anchorage-independent growth ability assay and flow cytometry analysis. The target of miR-208 was determined by western blotting analysis, luciferase reporter assay and real-time PCR.

**Results:**

miR-208 was upregulated in ESCC cell lines and tissues. Overexpression of miR-208 in ESCC cells increased cell proliferation, tumorigenicity and cell cycle progression, whereas inhibition of miR-208 reduced cells proliferation, tumorigenicity and cell cycle progression. Additionally, SOX6 was identified as a direct target of miR-208. Ectopic expression of miR-208 led to downregulation of SOX6 protein, which resulted in the downregulation of p21, upregulation of cyclin D1 and phosphorylation of Rb.

**Conclusions:**

These results suggest that miR-208 represents a potential onco-miR and participates in ESCC carcinogenesis by suppressing SOX6 expression.

## Introduction

Esophageal cancer, one of the most common malignant tumors, is the eighth most common cancer and the sixth most common causes of cancer mortality in the world [1]. According to its pathological characteristics, it has two main subtypes, ESCC and esophageal adenocarcinoma (EAC) [1,2]. ESCC is the major histological type of esophageal cancer in developing countries [3]. Although advances have been made in the treatment of ESCC, including surgery, chemotherapy, radiation or a combination of these options, the prognosis of ESCC patients remains very poor, which the overall 5-year survival rate of patient after surgery is only about 14-22% [3,4]. Some oncogenic and tumor suppressive factors have been reported to be associated with ESCC progression; however, few of them were specific and conclusive [5,6]. Further information on the biological behavior involved in esophageal cancer initiation and progression, especially in ESCC, is important for the development of effective diagnostic methods and therapeutic strategies.

MiRNAs, a class of small non-coding RNAs of 20–22 nucleotides, are involved in multiple biological processes, such as cell differentiation, proliferation, oncogenesis, angiogenesis and cell invasion [7-9]. MiRNAs play essential roles during human cancer progression by targeting the 3′ untranslated region (3′-UTR) of mRNAs in a sequence-specific manner for translational repression or degradation [10,11]. In view of the close relationship between miRNAs and the biological progression of multiple cancers, miRNAs are presently considered as potential novel targets for anti-cancer therapies [9-12].

SOX6, a member of the D sub family of the sex determining region Y (SRY)-box-related transcription factors, contains a conserved high-mobility-group (HMG) DNA-binding domain and plays important roles in biological progression [13,14]. SOX6 has been reported to play a tumor-suppressive function in certain tumors. It is frequently downregulated and significantly associated with better prognosis in primary ESCC and hepatocellular carcinoma [15,16].

In this study, we found that miR-208 is upregulated in ESCC cell lines and tissues. We verified a functional role for miR-208 in ESCC cell proliferation, tumorigenicity and cell cycle regulation. Furthermore, our data indicated that SOX6 mRNA is a target of miR-208 and that SOX6 is essential for the regulation of miR-208 in ESCC cells *in vitro*. Our results suggest that miR-208 may promote cell proliferation, tumorigenicity and cell cycle progression in ESCC through the SOX6-mediated signaling pathway.

## Material and methods

### Cell culture

Primary culture of normal esophageal epithelial cells (NEEC) was established from fresh specimens of the adjacent noncancerous esophageal tissue, which is over 5 cm from the cancerous tissue, according to a previous report [17]. The ESCC cell lines, including Kyse140, Kyse30, Kyse510, Kyse520, Eca109, TE-1, Kyse410, Kyse180, EC18, HKESC1 and 108CA, were grown in the Dulbecco’s modified Eagle’s medium (DMEM, Invitrogen, Carlsbad, CA, USA) supplemented with 10% fetal bovine serum (FBS) (HyClone, Logan, UT, USA), 100 units of penicillin and 100 units of streptomycin at 37°C in a 5% CO_2_ atmosphere in a humidified incubator.

### Tissue specimens

For the use of the clinical materials for research purposes, prior patient consent and approval from the Institutional Research Ethics Committee were obtained. This study was conducted on 10 pairs of snap-frozen ESCC tumor and matched normal tissues from adjacent regions, which were histopathologically diagnosed at the First Affiliated Hospital of Sun Yat-sen University from 2001 to 2006. The 10 ESCC tissues and the matched adjacent noncancerous esophageal tissues were frozen and stored in liquid nitrogen until further use.

### Generation of stably engineered cell lines

The miR-208 expression plasmid was generated by cloning the genomic pre-miR-208 gene, with 300-bp on each flanking side, into retroviral transfer plasmid pMSCV-puro (Clontech Laboratories Inc., Moutain View, CA, USA) to generate plasmid pMSCV-miR-208. The non-targeting control microRNA (negative control mimic), which are designed computationally to have no perfect seed-sequence matches to the transcriptome, was subcloned into retroviral transfer plasmid pMSCV to generate the plasmid pMSCV-NC. pMSCV-miR-208 or pMSCV-NC was cotransfected with the PIK packaging plasmid into 293FT cells using the standard calcium phosphate transfection method [18]. Thirty-six hours after cotransfection, supernatants were collected and incubated with cells to be infected for 24 hours in the presence of polybrene (2.5 μg/ml). After infection, puromycin (1.5 μg/ml) was used to select stably transduced cells over a 10-day period.

### RNA extraction, reverse transcription (RT) and real-time PCR

Total cellular RNA was extracted using the Trizol reagent (Invitrogen, Carlsbad, CA, USA) according to the manufacturer’s instruction. cDNAs were amplified and quantified in an ABI Prism 7500 Sequence Detection System (Applied Biosystems, Foster City, CA, USA) using dye SYBR Green I (Molecular Probes, Invitrogen). The primers selected were as follows:

*p21* forward: 5′-CATGGGTTCTGACGGACAT -3′,

*p21* reverse: 5′- AGTCAGTTCCTTGTGGAGCC -3′;

*Cyclin D1* forward: 5′-AACTACCTGGACCGCTTCCT -3′,

*Cyclin D1* reverse: 5′-CCACTT GAGCTTGTTCACCA-3′.

Expression levels of genes were normalized to that of the housekeeping gene *GAPDH* as the control (*GAPDH* forward primer, 5′-GACTCATGACCACAGTCCA TGC-3′; reverse primer, 3′-AGAGGCAGGGATGATGTTCTG-5′), and calculated as 2^-[(Ct^^of p21, *CyclinD1*) – (Ct^^of *GAPDH*)]^, where C_t_ represents the threshold cycle for each transcript. The expression of the miRNA was defined based on the threshold cycle (Ct), and relative expression levels were calculated as 2^-[(Ct of miR-208) – (Ct of U6)]^ after normalization with reference to expression of U6 small nuclear RNA.

### Western blotting

Western blotting analysis was performed according to standard methods, as previously described [19]. The membranes were probed with polyclonal rabbit antibodies against anti-SOX6 (1:500; Abcam, Cambridge, MA, USA), anti-p21, anti-cyclinD1 and anti-Rb, anti-phosphorylated Rb (1:1,000; Cell Signaling, Danvers, MA, USA). The membranes were stripped and re-probed with an anti-α-Tubulin mouse monoclonal antibody (1:1,000; Sigma, Saint Louis, MO, USA) as a loading control.

### Plasmid, oligonucleotides, siRNA and transfection

The region of the human SOX6 3′-UTR, from 1 to 1100 bp, generated by PCR amplification from DNA of the Eca109 cells, was cloned into vector pGL3 (Promega, Madison, WI, USA). The primers selected were as follows: SOX6-3′UTR-wt-up: 5′- GCCCCGCGGTGGCTCCACAATTACATCAGC -3′, SOX6-3′UTR-wt-dn: 3′- GCCCTGCAGCATAAAATCACTATGTACACAGGA -5′. The miR-208 mimic, miR-208 inhibitor and negative control (NC) were purchased from RiboBio (RiboBio Co. Ltd, Guangzhou, Guangdong, China). For depletion of SOX6, the siRNA was synthesized and purified by RiboBio. The SOX6 siRNA sequences used were: CGGGAAACTGTCCTCCATAAA. Transfection of oligonucleotides and siRNA were performed using the Lipofectamine 2000 reagent (Invitrogen, Carlsbad, CA) according to the manufacturer’s instruction.

### Luciferase assay

ESCC cells (3.5 × 10^4^) were seeded in triplicate in 24-well plates and allowed to settle for 12 h. One hundred nanograms of pGL3-SOX6-luciferase plasmid was transfected into ESCC cells using the Lipofectamine 2000 reagent (Invitrogen). Medium was replaced after 6 h, and luciferase and renilla signals were measured 48 h after transfection using the Dual Luciferase Reporter Assay Kit (Promega) according to the manufacturer’s protocol. Three independent experiments were performed and the data were presented as the mean ± SD.

### 3-(4, 5-Dimethyl-2-thiazolyl)-2, 5-diphenyl-2H-tetrazolium bromide (MTT) assay

Kyse30 and Kyse410 cells, seeded on 96-well plates, were stained at the indicated time points with 100 μl sterile MTT dye (0.5 mg/ml, Sigma) for 4 h at 37ºC, followed by removal of the culture medium and addition of 150 μl dimethyl sulfoxide (Sigma). The absorbance was measured at 570 nm, with 655 nm as the reference wavelength. All experiments were performed in triplicate.

### Colony formation assay

Cells were plated on a 6-well plate (0.5 × 10^3^ cells per well) and cultured for 10 days. The colonies were stained with 1.0% crystal violet for 1 min after fixation with 10% formaldehyde for 5 min. The experiment was performed independently three times for each cell line.

### Anchorage-independent growth ability assay

One thousand cells were trypsinized and suspended in 2 ml complete medium plus 0.33% agar (Sigma). The agar-cell mixture was plated on top of a bottom layer comprising 0.66% complete medium agar mixture. After 10 days, colony sizes were measured with an ocular micrometer and colonies greater than 0.1 mm in diameter were counted. The experiment was performed for independently three times for each cell line.

### Flow cytometry analysis

All cells in a culture dish were harvested by trypsinization, washed in ice-cold PBS, and fixed in 80% ice-cold ethanol. Before staining, the cells were pelleted in a cooled centrifuge and resuspended in cold PBS. Bovine pancreatic RNAase (Sigma) was added at a final concentration of 2 μg/ml, and cells were incubated at 37°C for 30 min, followed by incubation in 20 μg/ml propidium iodide (Sigma) for 20 min at room temperature. 20,000 cells were analyzed on a flow cytometer (FACSCalibur; BD Biosciences, Bedford, MA, USA).

### Statistical analysis

Student’s *t* test was used to evaluate the significant difference of two groups of data in all the pertinent experiments. A *P* value <0.05 (using a two-tailed paired *t* test) was considered significantly different for two groups of data.

## Results

### miR-208 expression is elevated in ESCC cell lines and tissues

**Figure 1:**
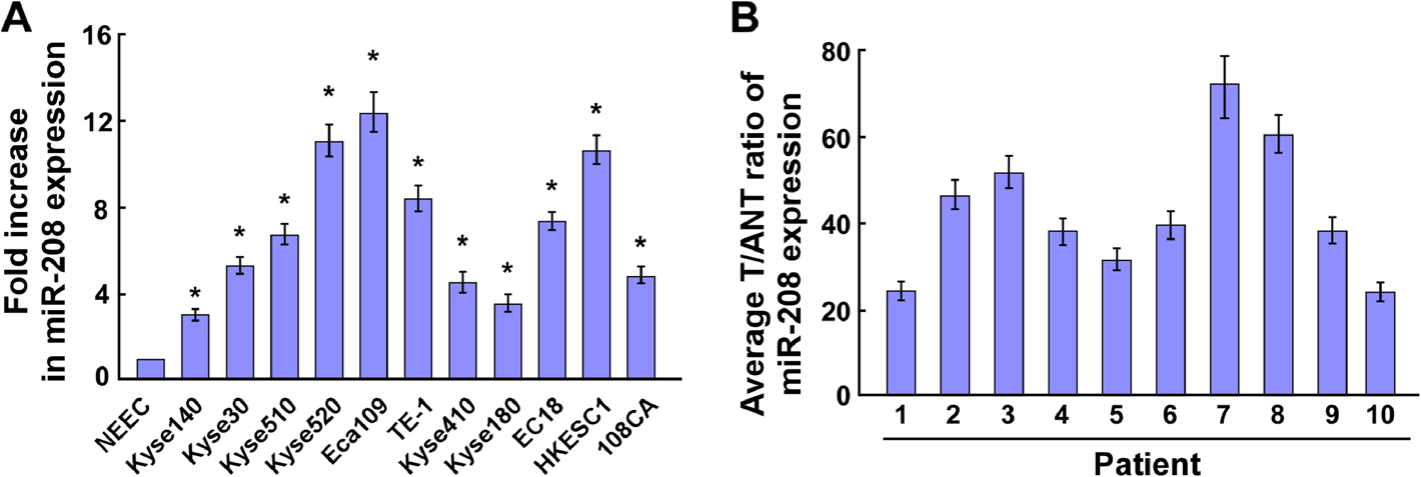
**Expression of miR-208 is increased in ESCC cell lines and tissues. A**. Real-time PCR analysis of miR-208 expression in normal esophageal epithelial cells (NEEC) and esophageal squamous carcinoma cells, including Kyse140, Kyse30, Kyse510, Kyse520, Eca109, TE-1, Kyse410, Kyse180, EC18, HKESC1 and 108CA. Transcript levels were normalized using U6 expression. **B**. The expression of miR-208 was examined in 10 paired cancerous tissues (T) and their adjacent noncancerous esophageal tissues (ANT). The average miR-208 expression was normalized using U6 expression. Each bar represents the mean ± SD of three independent experiments. *P <0.05.

Real-time PCR analysis revealed that miR-208 expression was markedly increased in all eleven ESCC cell lines, including Kyse140, Kyse30, Kyse510, Kyse520, Eca109, TE-1, Kyse410, Kyse180, EC18, HKESC1 and 108CA, compared with that in NEEC (Figure 1A). Moreover, comparative analysis revealed that miR-208 was significantly overexpressed in 10 pairs of cancerous tissues compared with the adjacent noncancerous esophageal tissues (Figure 1B). Collectively, these results suggested that miR-208 was upregulated in ESCC.

### Ectopic expression of miR-208 enhances proliferation of ESCC cells

**Figure 2:**
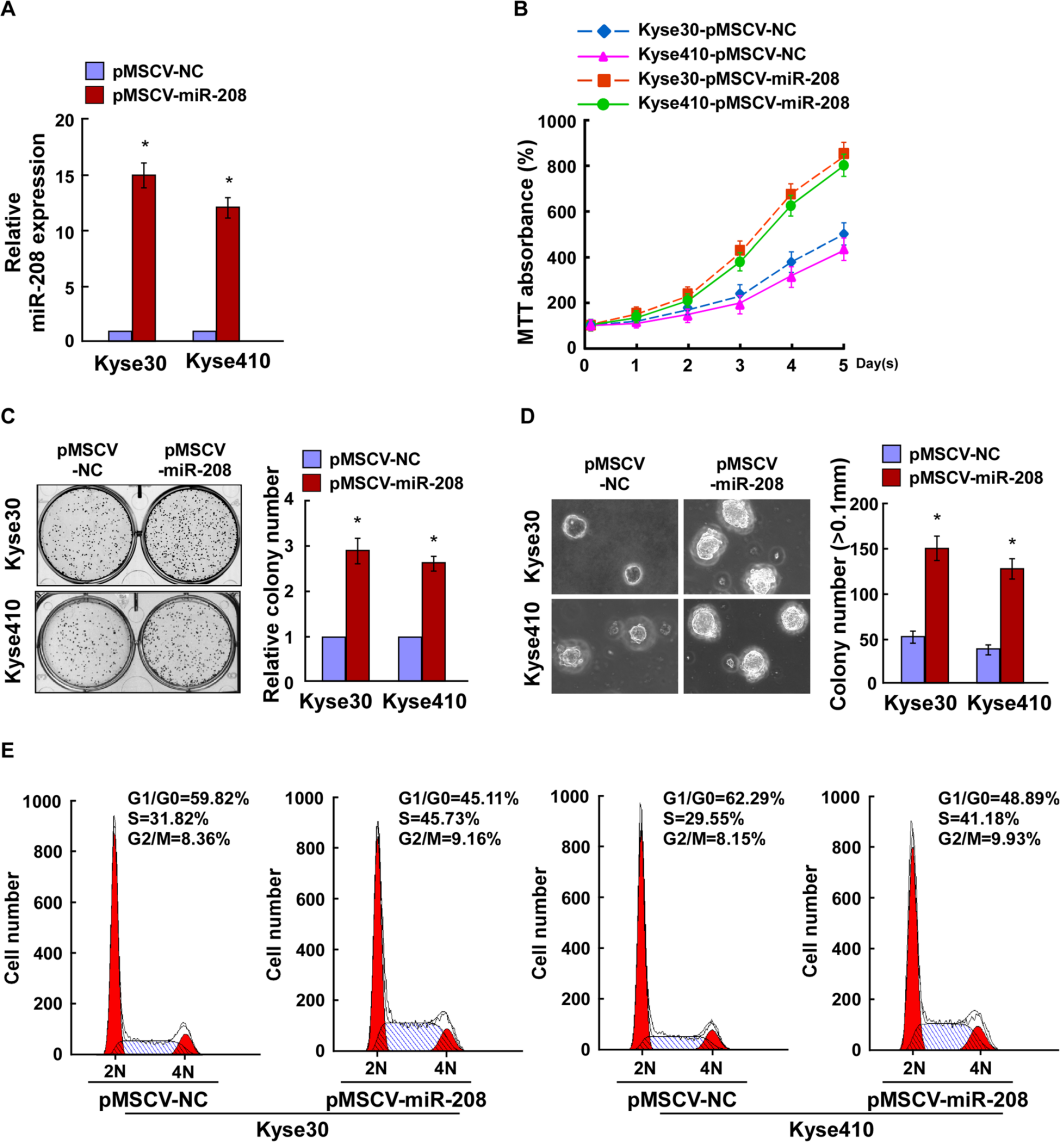
**Upregulation of miR-208 promotes the proliferation ability of ESCC cells. A**. Real-time PCR analysis of miR-208 expression in Kyse30 and Kyse410 cells stably expressing miR-208 and in control cells. **B**. Effects of ectopic miR-208 on the proliferation of Kyse30 and Kyse410 cells lines analyzed by the MTT assay. **C**. Representative micrographs (left) and quantification (right) of crystal violet stained cell colonies formed by the indicated ESCC cell lines, 10 days after inoculation. **D**. Effects of ectopic miR-208 on the tumorigenicity of Kyse30 and Kyse410 cell lines, as determined by an anchorage-independent growth ability assay. Colonies larger than 0.1 mm were scored. **E**. Effects of miR-208 overexpression on the cell cycle progression of ESCC cells measured by flow cytometry analysis. Each bar represents the mean ± SD of three independent experiments. *P <0.05.

To investigate the effect of miR-208 on the development and progression of ESCC, Kyse30 and Kyse410 ESCC cells, which were with medium-level of miR-208 expression, stably overexpressing miR-208 were established (Figure 2A). The MTT and colony formation assays showed that overexpression of miR-208 dramatically increased the growth rate of both ESCC cell lines compared with that of control cells (Figure 2B and C). Importantly, the anchorage-independent growth assay revealed that both Kyse30-miR-208 and Kyse410-miR-208 cells showed more and larger-sized colonies than their corresponding control cells (Figure 2D). Moreover, we analyzed the cell cycle of Kyse30-miR-208 and Kyse410-miR-208 cells by flow cytometry, which showed a significant decrease in the percentage of cells in G1/G0 phase and an increase in the percentage of cells in S phase (Figure 2E). All these results suggested that upregulation of miR-208 promoted the proliferation and tumorigenicity of ESCC cells.

### Inhibition of miR-208 reduces proliferation of ESCC cells

**Figure 3:**
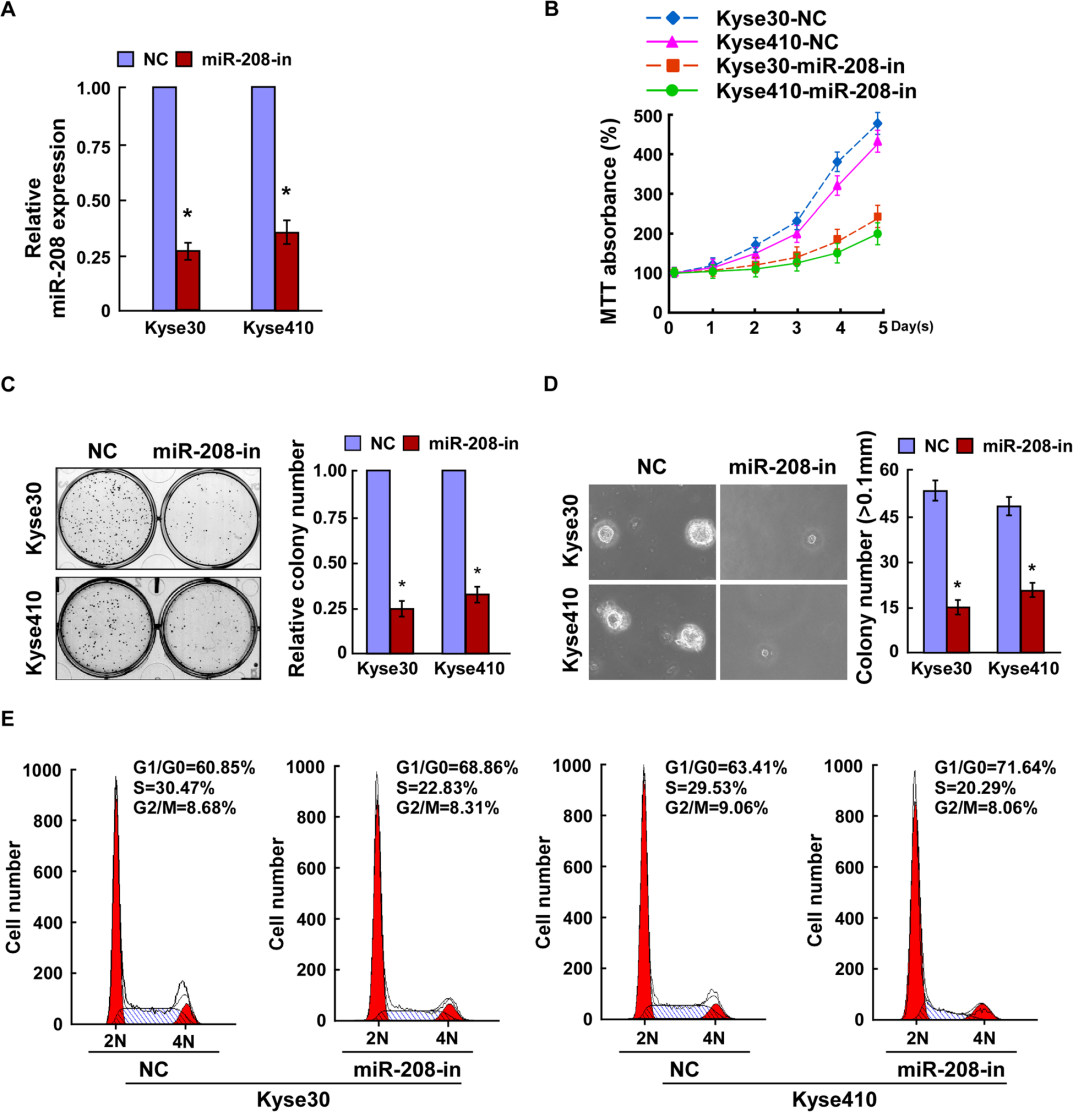
**Inhibition of miR-208 reduces the proliferation of ESCC. A**. Real-time PCR analysis of miR-208 expression in Kyse30 and Kyse410 cells transfected with miR-208 inhibitor. **B**. The proliferation ability of ESCC cells Kyse30 and Kyse410 transfected with miR-208 inhibitor or NC measured by the MTT assay. **C**. Representative micrographs (left) and quantification (right) of crystal violet stained cell colonies formed by indicated ESCC cell lines, 10 days after inoculation. **D**. The tumorigenicity of ESCC cells Kyse30 and Kyse410 transfected with miR-208 inhibitor or NC measured, as by an anchorage-independent growth ability assay. Colonies larger than 0.1 mm were scored. **E**. Flow cytometry analysis of indicated ESCC cells transfected with miR-208-inhibitor or NC. Each bar represents the mean ± SD of three independent experiments. *P <0.05.

To further test whether endogenous miR-208 helps to sustain the proliferative property of ESCC cells, loss-of-function studies using a miR-208 inhibitor were used to further investigate whether endogenous miR-208 helps to maintain the proliferative properties of ESCC cells. As shown in Figure 3A, B and C, suppression of miR-208 by transfection with the miR-208 inhibitor significantly decreased the growth rate of both ESCC cell lines as compared with that of NC transfected cells. The anchorage-independent growth assay revealed that both Kyse30-miR-208-inhibitor and Kyse410-miR-208-inhibitor cells formed fewer and smaller-sized colonies than their corresponding negative control cells, indicating the inhibitory function of miR-208 inhibitor on ESCC tumorigenicity (Figure 3D). Additionally, flow cytometry showed a significant increase in the percentage of cells in G1/G0 phase and a decrease in the percentage of cells in S phase in Kyse30-miR-208-inhibitor and Kyse410-miR-208-inhibitor cells compared with NC transfected cells (Figure 3E). These results suggested that downregulation of miR-208 could suppress the proliferation and tumorigenicity of ESCC cells.

### SOX6 is a direct target of miR-208 in ESCC cells

**Figure 4:**
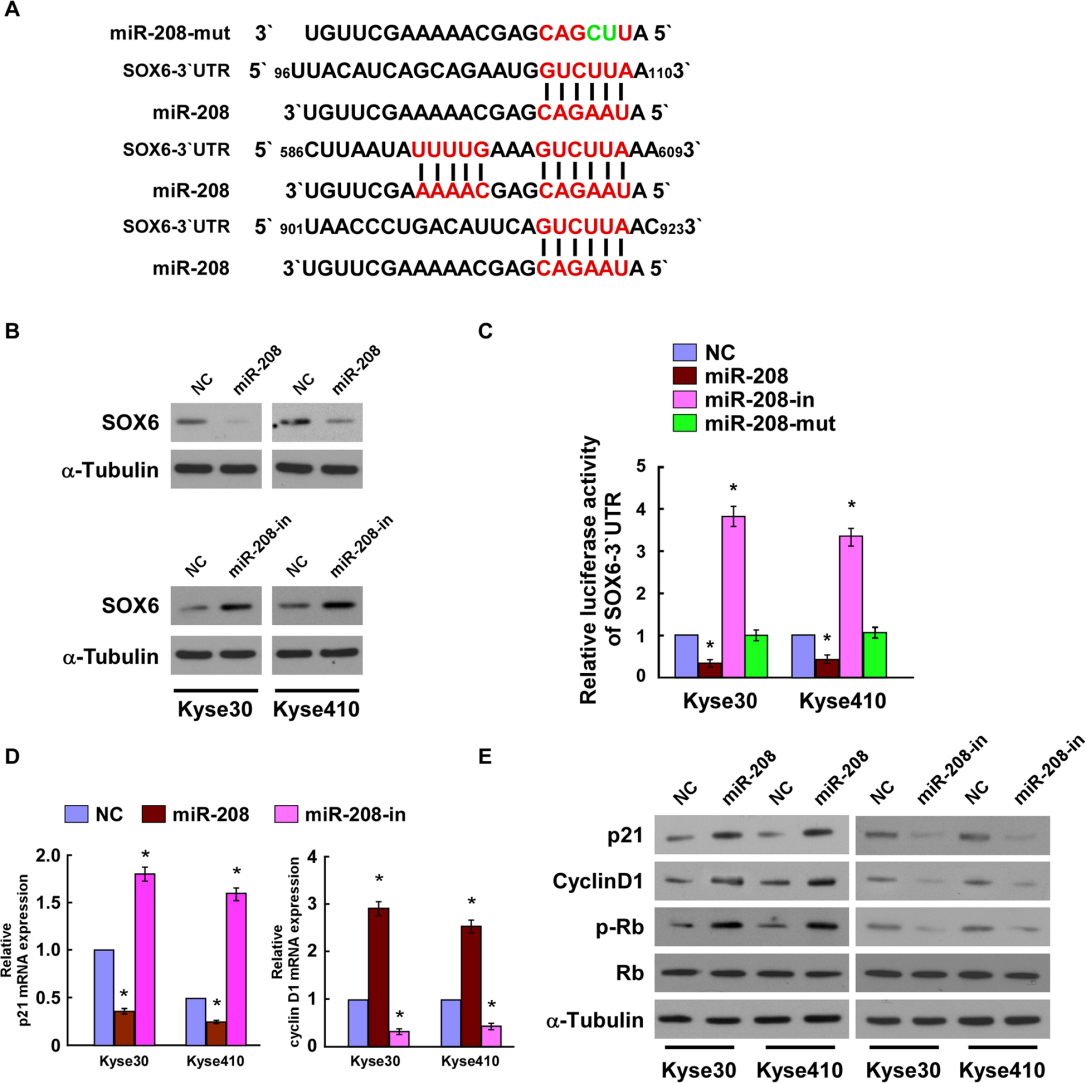
**miR-208 directly targets the 3′-UTR of *SOX6 *mRNA. A**. Schematic representation of the miR-208 target sites in the 3′-UTR of *SOX6* mRNA and a miR-208 mutant containing two altered nucleotides in the seed sequence (miR-208-mut). **B**. The expression levels of SOX6 protein in Kyse30 and Kyse410 cells overexpressing or suppressing miR-208, by western blotting 48 hours after transfection. α-Tubulin served as the loading control. **C**. Luciferase assay of indicated cells transfected with the pGL3-SOX6-3′UTR reporter. **D**. Real-time PCR analysis of SOX6 downstream genes mRNA expression in indicated ESCC cells. **E**. Expression of p21, CyclinD1, phosphorylated pRb, and Rb protein as measured by western blotting in indicated ESCC cells. α-Tubulin served as the loading control. Each bar represents the mean ± SD of three independent experiments. *P <0.05.

A previous study showed that SOX6 is a tumor-suppressor and plays an important role in ESCC progression [1]. Using publicly available algorithms (TargetScan, Pictar, miRANDA), we found that SOX6 was a potential target of miR-208 (Figure 4A). As predicted, western blotting analysis revealed that SOX6 expression decreased in the Kyse30 and Kyse410 ESCC cells ectopic expressing miR-208 and increased in cells suppressing miR-208 (Figure 4B). To examine whether miR-208 mediated-SOX6 downregulation was through the 3′-UTR of SOX6, the SOX6- 3′-UTR fragment, containing three miR-208 binding sites, was subcloned into a pGL3 luciferase reporter vector. The results of the luciferase reporter assay showed that overexpression of miR-208 decreased, and suppression of miR-208 increased, the luciferase activity of the SOX6 3′-UTR-luciferase reporter, whereas a miR-208 with mutated seed sequence failed to show an inhibitory effect on the luciferase activity (Figure 4C). Meanwhile, we observed that the mRNA of the SOX6 downstream gene, *p21* was significantly downregulated, and that of *Cyclin D1* was significantly upregulated, by miR-208 (Figure 4D). Moreover, the expression level of the p21 protein was downregulated, Cyclin D1 was upregulated, and phosphorylated Rb was increased in miR-208 overexpressing cells compared with the negative control cells. In contrast, the expression level of p21 was upregulated, Cyclin D1 was downregulated, and Rb phosphorylation was decreased in cells transfected with the miR-208 inhibitor (Figure 4E). Collectively, our results suggested that SOX6 was a *bona fide* target of miR-208.

### SOX6 suppression is critical for miR-208-induced cell proliferation in ESCC

**Figure 5:**
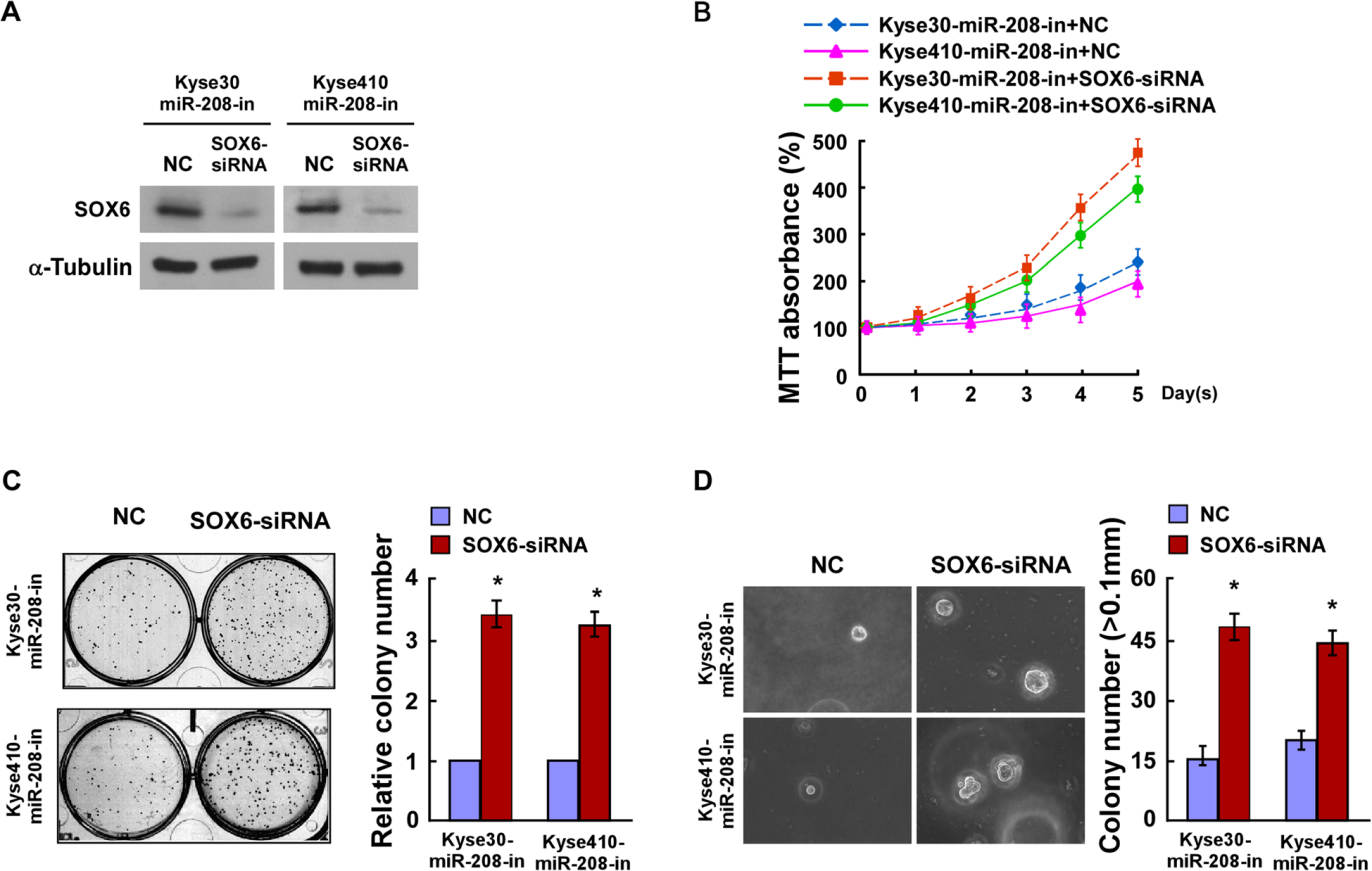
**miR-208 promotes ESCC progression by inhibiting SOX6. A**. The expression levels of SOX6 in Kyse30-miR-208-inhibitor and Kyse410-miR-208-inhibitor cells that were transfected with SOX6-siRNA, as measured by western blotting. α-Tubulin served as the loading control. **B**. The growth rates in SOX6-silenced cells as indicated by the MTT assay. **C**. Representative micrographs (left) and quantification (right) of crystal violet stained cell colonies formed by indicated ESCC cell lines 10 days after inoculation. **D**. Representative images (left) and quantification (right) of colony numbers of indicated cells determined by an anchorage-independent growth assay. Colonies larger than 0.1 mm in diameter were scored. Error bars represent mean ± SD from three independent experiments. *P < 0.05.

To investigate the effect of SOX6 reduction on ESCC progression, we repressed endogenous SOX6 expression using a SOX6-specific siRNA (Figure 5A). The MTT assay and the colony formation assay both showed that silencing SOX6 in miR-208 inhibitor transfected cells increased the growth rate of the cells (Figure 5B and C). The anchorage-independent growth assay showed similar results (Figure 5D). All the results suggested that further silencing SOX6 expression in Kyse30-miR-208-inhibitor and Kyse410-miR-208-inhibitor cells could reverse the inhibitory effect of the miR-208 inhibitor on ESCC cells proliferation. These data confirmed that miR-208 promoted ESCC cells proliferation and tumorigenicity by repressing endogenous SOX6 expression and that SOX6 plays important role in miR-208-mediated cell proliferation.

## Discussion

In the current study, we demonstrated that miR-208 is upregulated in ESCC cell lines and tissues. Overexpression of miR-208 promotes the proliferation and tumorigenicity of ESCC cells, probably through post-translationally downregulating SOX6 expression by targeting its mRNA 3′- UTR. The negative regulation of SOX6 by miR-208 leads to upregulation of p21, downregulation of cyclinD1, and Rb phosphorylation. We demonstrated that miR-208 might play essential role via the SOX6-mediated pathway during ESCC progression.

Recent studies showed that miRNAs regulated various cellular pathways by affecting the expression of multiple target genes [8,20]. The expression of miRNAs might contribute to human carcinogenesis and cancer progression, and are considered as potential novel targets for cancer diagnosis and therapy [21-24]. miR-208 has been identified as a myomiR. It is specifically expressed at much higher levels in cardiac tissue and is dysregulated in various cardiovascular diseases [25-27]. Inhibition of miR-208 improved cardiac function and patient survival during heart failure [27,28]. However, the expression and function of miR-208 in cancers remain unknown. For the first time, we have demonstrated that miR-208 expression is correlated with ESCC progression and might play an important role during ESCC development. It has also been reported that miR-208 downregulates the Ets1 proto-oncogene, which is closely involved in the regulation of cell proliferation, differentiation, metastasis, apoptosis, and angiogenesis, by targeting the Ets1 mRNA 3′-UTR [29]. Nevertheless, the expression level of miR-208 in esophageal cancer or other cancers and its clinical relevance, require further study.

Systematic reporter studies have shown that functional regulation by miRNAs is highly sensitive to base pair mismatches within nucleotides 2–8 of the miRNA, which have been defined as the seed region [30]. The functional importance of seed region complementarity as the major determinant of miRNA targeting has well established [31], which the microRNAs with mutation in the seed sequences could not genetically match with target genes and fail to inhibit the target genes expression [8,32]. In the current study, we found that miR-208 negatively regulated SOX6 expression by targeting SOX6 3′ untranslated region (3′-UTR) in the sequence-specific manner. Consistently, miR-208 with mutated seed sequence failed to show an inhibitory effect on the luciferase activity of SOX6. SOX6 is reported to have a tumor-suppressive function in tumors [15,16]. SOX6 suppressed ESCC cells proliferation and cell motility, and inhibited tumor formation. SOX6 also inhibits cell cycle G1/S phage transition by upregulating p53 and p21^WAF1/CIP1^, and by downregulating cyclin D1/CDK4, cyclin A and β–catenin [14,15]. In our study, downregulation of SOX6 by miR-208 led to downregulation of p21, upregulation of cyclin D1, and induced phosphorylation of Rb, resulting in the promotion of cell proliferation and cell cycle progression.

These data suggested that overexpression of miR-208 favored ESCC progression. Most patients with ESCC are diagnosed at an advanced stage with lymph node (LN) metastasis, resulting in a poor prognosis [33]. Therefore, a better understanding of the development of LN metastasis may lead to therapeutic improvements for ESCC patients. SOX6 is closely associated with LN metastasis of ESCC [15]. Whether miR-208 is involved in SOX6-associated LN metastasis needs further study.

In summary, we describe, for the first time, that miR-208 plays an important role in ESCC development and progression. The results reveal that miR-208 is upregulated in ESCC cell lines and tissues. Furthermore, miR-208 overexpression promotes cell proliferation and tumorigenicity in human ESCC cell lines *in vitro*. Additionally, we identified SOX6 mRNA as a direct and functional target of miR-208. Finally, we revealed that SOX6 suppression is essential for miR-208-induced cell proliferation in ESCC. Based on these results, we propose that miR-208 might be used as a therapeutic agent for ESCC. Further study is required to identify the clinical relevance and utility of miR-208 in ESCC diagnosis and therapy.

## Competing interests

All authors declare that they have no competing interests.

## Authors’ contributions

HL, YG and JY participated in the design of study. HL, DZ, BZ, LL, JO and WC performed experimental work. HL, DZ, BZ and SX performed the statistical analysis and helped to draft the manuscript. YG and JY provided administrative support and funded experiments. All authors read and approved the final manuscript.
